# Supplementary material for: Urate-lowering effects of polyphenolic compounds in animal models: systematic review and meta-analysis
Source: PeerJ. 2025 Aug 11;13:e19731. doi: 10.7717/peerj.19731 (PMC12352422; doi:10.7717/peerj.19731)
Supplement: Supplemental Information 2 [file peerj-13-19731-s002.docx]

**Supplementary materials 1: Search strategy**

**PubMed**

(((((("Resveratrol"[Mesh]) OR (((((((((((((((3,4',5-Stilbenetriol) OR (3,5,4'-Trihydroxystilbene)) OR (3,4',5-Trihydroxystilbene)) OR (trans-Resveratrol)) OR (trans Resveratrol)) OR (Resveratrol-3-sulfate)) OR (Resveratrol 3 sulfate)) OR (SRT 501)) OR (SRT-501)) OR (SRT501)) OR (cis-Resveratrol)) OR (cis Resveratrol)) OR (Resveratrol, (Z)-)) OR (trans-Resveratrol-3-O-sulfate)) OR (trans Resveratrol 3 O sulfate))) OR (("Chlorogenic Acid/pharmacokinetics"[Mesh]) OR ((((Acid, Chlorogenic) OR (3-Caffeoylquinic Acid)) OR (3 Caffeoylquinic Acid)) OR (Acid, 3-Caffeoylquinic)))) OR ("punicalagin" [Supplementary Concept])) OR (("ferulic acid" [Supplementary Concept]) OR (((((((((trans-ferulic acid) OR (4-hydroxy-3-methoxycinnamic acid)) OR (ferulic acid, (E)-isomer)) OR (3-(4-hydroxy-3-methoxyphenyl)-2-propenoic acid)) OR (sodium ferulate)) OR (ferulic acid, monosodium salt)) OR (ferulic acid, (Z)-isomer)) OR (cis-ferulic acid)) OR (8,8'-diferulic acid)))) OR (("bergenin" [Supplementary Concept]) OR ((bergenin, (2alpha,3beta,4alpha,4aalpha,10bbeta)-isomer, sodium salt) OR (bergenin hydrate)))) AND (("Uric Acid"[Mesh]) OR (((((((((((((((((((((((Acid, Uric) OR (2,6,8-Trihydroxypurine)) OR (Trioxopurine)) OR (Ammonium Acid Urate)) OR (Acid Urate, Ammonium)) OR (Urate, Ammonium Acid)) OR (Potassium Urate)) OR (Urate, Potassium)) OR (Sodium Urate Monohydrate)) OR (Monohydrate, Sodium Urate)) OR (Urate Monohydrate, Sodium)) OR (Monosodium Urate)) OR (Urate, Monosodium)) OR (Monosodium Urate Monohydrate)) OR (Monohydrate, Monosodium Urate)) OR (Urate Monohydrate, Monosodium)) OR (Sodium Acid Urate)) OR (Acid Urate, Sodium)) OR (Urate, Sodium Acid)) OR (Sodium Acid Urate Monohydrate)) OR (Sodium Urate)) OR (Urate, Sodium)) OR (Urate)))

**Embase**

1. 'chlorogenic acid'/exp OR '1, 3, 4, 5 tetrahydroxycyclohexanecarboxylic acid 3 (3, 4 dihydroxycinnamate)' OR '3 (3, 4 dihydroxycinnamoyl) quinic acid' OR '3 caffeoylquinic acid' OR '3 o caffeoylquinic acid' OR 'cis chlorogenic acid' OR 'trans chlorogenic acid' OR 'chlorogenic acid'

2. 'resveratrol'/exp OR '3, 4`, 5 stilbenetriol' OR '3, 4`, 5 trihydroxystilbene' OR '5 (4 hydroxystyryl) benzene 1, 3 diol' OR 'srt 501' OR 'srt501' OR 'trans resveratrol' OR 'trans-resveratrol' OR 'resveratrol'

3. 'punicalagin'/exp

4. 'ferulic acid'/exp OR '3 (4 hydroxy 3 methoxyphenyl) propenoic acid' OR '3 methoxy 4 hydroxycinnamic acid' OR '4 hydroxy 3 methoxycinnamic acid' OR 'ferula acid' OR 'ferulate sodium' OR 'sodium ferulate' OR 'ferulic acid'

5. 'bergenin'/exp OR 'bergenin'

6.'uric acid'/exp OR '2, 6, 8 trioxypurine' OR 'uric acid dihydrate' OR 'uric acid'

7. ('chlorogenic acid'/exp OR '1, 3, 4, 5 tetrahydroxycyclohexanecarboxylic acid 3 (3, 4 dihydroxycinnamate)' OR '3 (3, 4 dihydroxycinnamoyl) quinic acid' OR '3 caffeoylquinic acid' OR '3 o caffeoylquinic acid' OR 'cis chlorogenic acid' OR 'trans chlorogenic acid' OR 'chlorogenic acid') OR ('resveratrol'/exp OR '3, 4`, 5 stilbenetriol' OR '3, 4`, 5 trihydroxystilbene' OR '5 (4 hydroxystyryl) benzene 1, 3 diol' OR 'srt 501' OR 'srt501' OR 'trans resveratrol' OR 'trans-resveratrol' OR 'resveratrol') OR 'punicalagin'/exp OR ('ferulic acid'/exp OR '3 (4 hydroxy 3 methoxyphenyl) propenoic acid' OR '3 methoxy 4 hydroxycinnamic acid' OR '4 hydroxy 3 methoxycinnamic acid' OR 'ferula acid' OR 'ferulate sodium' OR 'sodium ferulate' OR 'ferulic acid') OR ('bergenin'/exp OR 'bergenin')

8.('uric acid'/exp OR '2, 6, 8 trioxypurine' OR 'uric acid dihydrate' OR 'uric acid') AND (('chlorogenic acid'/exp OR '1, 3, 4, 5 tetrahydroxycyclohexanecarboxylic acid 3 (3, 4 dihydroxycinnamate)' OR '3 (3, 4 dihydroxycinnamoyl) quinic acid' OR '3 caffeoylquinic acid' OR '3 o caffeoylquinic acid' OR 'cis chlorogenic acid' OR 'trans chlorogenic acid' OR 'chlorogenic acid') OR ('resveratrol'/exp OR '3, 4`, 5 stilbenetriol' OR '3, 4`, 5 trihydroxystilbene' OR '5 (4 hydroxystyryl) benzene 1, 3 diol' OR 'srt 501' OR 'srt501' OR 'trans resveratrol' OR 'trans-resveratrol' OR 'resveratrol') OR 'punicalagin'/exp OR ('ferulic acid'/exp OR '3 (4 hydroxy 3 methoxyphenyl) propenoic acid' OR '3 methoxy 4 hydroxycinnamic acid' OR '4 hydroxy 3 methoxycinnamic acid' OR 'ferula acid' OR 'ferulate sodium' OR 'sodium ferulate' OR 'ferulic acid') OR ('bergenin'/exp OR 'bergenin'))

**Cochrane Library**

1.MeSH descriptor: [Resveratrol] explode all trees

2.（3,5,4' Trihydroxystilbene）OR（3,4',5 Trihydroxystilbene）OR (trans Resveratrol) OR (trans Resveratrol) OR (Resveratrol 3 sulfate) OR (Resveratrol 3 sulfate) OR( SRT 501) OR (SRT 501) OR (SRT501)OR(cis Resveratrol) OR (cis Resveratrol) OR (Resveratrol, (Z) ) OR (trans Resveratrol 3 O sulfate) OR (trans Resveratrol 3 O sulfate)

3.MeSH descriptor: [Chlorogenic Acid] explode all trees

4.（Acid, Chlorogenic） OR（3 Caffeoylquinic Acid） OR（3 Caffeoylquinic Acid）OR（Acid, 3 Caffeoylquinic）

5.MeSH descriptor: [] explode all trees

6.（trans ferulic acid）OR（4 hydroxy 3 methoxycinnamic acid）OR（ferulic acid, (E) isomer）OR（3 (4 hydroxy 3 methoxyphenyl) 2 propenoic acid）OR（sodium ferulate）OR（ferulic acid, (Z) isomer）OR（cis ferulic acid）OR（8,8' diferulic acid）

7.MeSH descriptor: [] explode all trees

8.(bergenin, (2alpha,3beta,4alpha,4aalpha,10bbeta) isomer, sodium salt)OR(bergenin hydrate)

9.MeSH descriptor: [] explode all trees

10.MeSH descriptor: [Uric Acid] explode all trees

11.(Acid, Uric) OR (2,6,8 Trihydroxypurine) OR (Trioxopurine) OR (Ammonium Acid Urate) OR (Acid Urate, Ammonium) OR (Urate, Ammonium Acid) OR (Potassium Urate) OR (Urate, Potassium) OR (Sodium Urate Monohydrate) OR (Monohydrate, Sodium Urate) OR (Urate Monohydrate, Sodium) OR (Monosodium Urate) OR (Urate, Monosodium) OR (Monosodium Urate Monohydrate) OR (Monohydrate, Monosodium Urate) OR (Urate Monohydrate, Monosodium) OR (Sodium Acid Urate) OR (Acid Urate, Sodium) OR (Urate, Sodium Acid) OR (Sodium Acid Urate Monohydrate) OR (Sodium Urate) OR (Urate, Sodium) OR (Urate)

12.1 OR 2 OR 3

13.10 OR 11

14.12 AND 13
