# Supplementary material for: Urate-lowering effects of polyphenolic compounds in animal models: systematic review and meta-analysis
Source: PeerJ. 2025 Aug 11;13:e19731. doi: 10.7717/peerj.19731 (PMC12352422; doi:10.7717/peerj.19731)
Supplement: Supplemental Information 3 [file peerj-13-19731-s003.docx]

**Table S1**. Subgroup analysis for each outcome measures.

| **Variables** | **No. of Trials** | **SMD [95%CI]** | ***P* value** | ***I^2^*(%)** | ***P*-heterogeneity** |
| --- | --- | --- | --- | --- | --- |
| **The effect of total polyphenols on SUA** |  |  |  |  |  |
| Animal types |  |  |  |  |  |
| Wistar rat | 10 | -1.84 [-3.18，-0.5] | 0.007 | 90 | 0.000 |
| SD rat | 20 | -1.47 [-1.90，-1.05] | 0.000 | 77 | 0.000 |
| Albino rat | 1 | -1.46 [-2.67，-0.25] | 0.003 | 44 | 0.169 |
| Swiss mice | 5 | -2.20 [-2.94，-1.46] | 0.000 | 27 | 0.220 |
| Km mice | 9 | -5.56 [-6.87，-4.25] | 0.000 | 92 | 0.000 |
| C57BL/6 mice | 4 | -1.38 [-2.29，-0.46] | 0.003 | 77 | 0.001 |
| 9SHR | 1 | -1.94 [-3.29，-0.60] | － | － | － |
| ICR mice | 1 | -1.57 [-2.73，-0.41] | － | － | － |
| Disease Models |  |  |  |  |  |
| Diabetes Mellitus | 9 | -1.69 [-2.42，-0.96] | 0.000 | 76 | 0.000 |
| Hepatic Disease | 1 | -3.29 [-4.68，-1.90] | 0.000 | 0 | 0.909 |
| Metabolic Disease | 20 | -2.54 [-3.16，-1.91] | 0.000 | 88 | 0.000 |
| Kidney Disease | 13 | -2.43 [-3.32，-1.54] | 0.000 | 91 | 0.000 |
| CVD | 3 | -1.79 [-3.24，-0.34] | 0.015 | 69 | 0.040 |
| Multisystem Disease | 5 | -2.39 [-3.89，-0.89] | 0.002 | 82 | 0.000 |
| **RES on SUA** |  |  |  |  |  |
| Animal types |  |  |  |  |  |
| Wistar rat | 6 | -2.68 [-5.16，-0.19] | 0.035 | 93 | 0.000 |
| SD rat | 14 | -1.41 [-1.81，-1.02] | 0 | 71 | 0 |
| Albino rat | 1 | -1.46 [-2.67，-0.25] | 0.003 | 44 | 0.169 |
| Swiss mice | 2 | -3.29 [-4.68，-1.90] | 0 | 0 | 0.909 |
| Km mice | 3 | -2.99 [-4.54，-1.44] | 0 | 84 | 0 |
| C57BL/6 mice | 3 | -2.07 [-4.25，0.11] | 0.062 | 88 | 0 |
| ICR mice | 1 | -1.57 [-2.73，-0.41] | － | － | － |
| Drug route |  |  |  |  |  |
| p.o | 11 | -2.38 [-3.68，-1.08] | 0.000 | 90 | 0.000 |
| i.g. | 16 | -1.82 [-2.32，-1.33] | 0.000 | 78 | 0.000 |
| i.p. | 3 | -1.35 [-1.90，-0.79] | 0.000 | 53 | 0.049 |
| Does |  |  |  |  |  |
| 0～25mg/kg | 23 | -2.14 [-2.87，-1.42] | 0.000 | 84 | 0.000 |
| 26mg/kg～50mg/kg | 9 | -2.72 [-3.44，-2.01] | 0 | 80 | 0.056 |
| 51mg/kg～100mg/kg | 7 | -1.19 [-1.89，-0.49] | 0.001 | 75 | 0.001 |
| 101mg/kg～300mg/kg | 3 | -0.81 [-1.26，-0.36] | 0 | 0 | 0.417 |
| 300mg/kg～1000mg/kg | 2 | -0.92 [-1.59，-0.26] | 0.007 | 0 | 0.359 |
| Intervention Duration |  |  |  |  |  |
| 0～2week | 10 | -1.31 [-1.70，-0.92] | 0.000 | 64 | 0.000 |
| 2week～4week | 4 | -2.48 [-3.91，-1.05] | 0.001 | 86 | 0 |
| 4week～8week | 10 | -2.69 [-4.13，-1.25] | 0 | 92 | 0 |
| 8week～12week | 4 | -2.05 [-3.33，-0.78] | 0.002 | 77 | 0.001 |
| 12week～24week | 2 | -1.86 [-4.82，1.11] | 0.22 | 84 | 0.013 |
| **CGA on SUA** |  |  |  |  |  |
| Animal types |  |  |  |  |  |
| Wistar rat | 1 | -3.98 [-5.82，-2.13] | 0.000 | 0 | 0.330 |
| SD rat | 1 | -4.38 [-6.39，-2.38] | － | － | － |
| Swiss mice | 3 | -1.85 [-2.61，-1.09] | 0 | 15 | 0.319 |
| Km mice | 3 | -2.11 [-2.87，-1.35] | 0 | 65 | 0.014 |
| Drug route |  |  |  |  |  |
| p.o | 3 | -2.36 [-3.24，-1.47] | 0.001 | 68 | 0.004 |
| i.g. | 4 | -2.30 [-3.36，-1.25] | 0.000 | 61 | 0.003 |
| i.p. | 1 | -2.41 [-3.72，-1.09] | 0.000 | 0 | 0.446 |
| Does |  |  |  |  |  |
| 0～25mg/kg | 6 | -2.00 [-2.66，-1.34] | 0.000 | 14 | 0.325 |
| 26mg/kg～50mg/kg | 3 | -1.15 [-1.80，-0.5] | 0 | 0 | 0.465 |
| 51mg/kg～100mg/kg | 4 | -2.51 [-3.44，-1.58] | 0 | 25 | 0.264 |
| 101mg/kg～300mg/kg | 4 | -3.81 [-4.85，-2.78] | 0 | 0 | 0.44 |
| 26mg/kg～50mg/kg | 3 | -1.15 [-1.80，-0.5] | 0 | 0 | 0.465 |
| Intervention Duration |  |  |  |  |  |
| 0～2week | 6 | -2.18 [-2.84，-1.52] | 0.000 | 57 | 0.012 |
| 2week～4week | 2 | -2.86 [-4.29，-1.42] | 0.000 | 64 | 0.041 |
| **FA on SUA** |  |  |  |  |  |
| Animal types |  |  |  |  |  |
| Wistar rat | 4 | -0.24 [-1.87，1.39] | 0.772 | 81 | 0.001 |
| SD rat | 5 | -0.52 [-2.01，0.97] | 0.491 | 85 | 0 |
| Km mice | 1 | -25.23 [-29.07，-21.39] | 0 | 0 | 0.777 |
| 9SHR | 1 | -1.94 [-3.29，-0.60] | － | － | － |
| Drug route |  |  |  |  |  |
| p.o | 7 | 0.08 [-1.38，1.55] | 0.912 | 87 | 0.000 |
| i.g. | 3 | -12.47 [-17.87，-7.06] | 0.000 | 96 | 0.000 |
| i.p. | 1 | -0.30 [-1.29，0.68] | － | － | － |
| Does |  |  |  |  |  |
| 26mg/kg～50mg/kg | 7 | -0.10 [-1.47，1.27] | 0.887 | 85 | 0.000 |
| 51mg/kg～100mg/kg | 4 | -3.94 [-9.10，1.22] | 0.134 | 96 | 0 |
| 101mg/kg～300mg/kg | 5 | -9.97 [-15.00，-4.95] | 0 | 96 | 0 |
| Intervention Duration |  |  |  |  |  |
| 0～2week | 2 | -18.93[-36.12，-1.74] | 0.030 | 98 | 0.000 |
| 2week～4week | 3 | 2.42 [-1.62，6.46] | 0.24 | 90 | 0 |
| 4week～8week | 3 | -1.01 [-3.27，1.26] | 0.382 | 85 | 0 |
| 8week～12week | 2 | -1.71 [-2.66，-0.76] | 0 | 0 | 0.624 |
| 12week～24week | 1 | -1.86 [-2.95，-0.77] | 0 | 48 | 0.165 |
| **PU on SUA** |  |  |  |  |  |
| Animal types |  |  |  |  |  |
| Km mice | 1 | -6.49 [-9.60，-3.39] | 0.000 | 79 | 0.008 |
| C57BL/6 mice | 1 | -0.87 [-1.40，-0.33] | 0.001 | 0 | 0.629 |
| Does |  |  |  |  |  |
| 26mg/kg～50mg/kg | 1 | -1.00 [-1.77，-0.24] | － | － | － |
| 51mg/kg～100mg/kg | 2 | -2.26 [-5.42，0.89] | 0.159 | 92 | 0.000 |
| 101mg/kg～300mg/kg | 2 | -7.93 [-9.98，-5.88] | 0.000 | 0 | 0.391 |
| Intervention Duration |  |  |  |  |  |
| 0～2week | 1 | -6.49 [-9.60，-3.39] | 0.000 | 79 | 0.008 |
| 4week～8week | 1 | -0.87 [-1.40，-0.33] | 0.001 | 0 | 0.629 |
| **The effect of total polyphenols on UUA** |  |  |  |  |  |
| Animal types |  |  |  |  |  |
| SD rat | 2 | 1.42 [-1.53，4.37] | 0.346 | 93 | 0.000 |
| Km mice | 4 | 2.90 [1.76，4.03] | 0.000 | 88 | 0.000 |
| Disease Models |  |  |  |  |  |
| Metabolic Disease | 4 | 1.21 [0.12，2.31] | 0.002 | 90 | 0.000 |
| Kidney Disease | 1 | 5.22 [1.63，8.82] | 0.004 | 65 | 0.093 |
| Multisystem Disease | 1 | 6.35 [4.77，7.93] | 0.000 | 0 | 0.486 |
| Types of Intervention |  |  |  |  |  |
| RES | 3 | 2.40 [0.23，4.57] | 0.030 | 94 | 0.000 |
| BER | 1 | 4.06 [2.63，5.48] | 0 | 53 | 0.117 |
| CGA | 1 | 0.52 [0.05，0.99] | 0.031 | 0 | 0.893 |
| FA | 1 | 5.22 [1.63，8.82] | 0.004 | 65 | 0.093 |


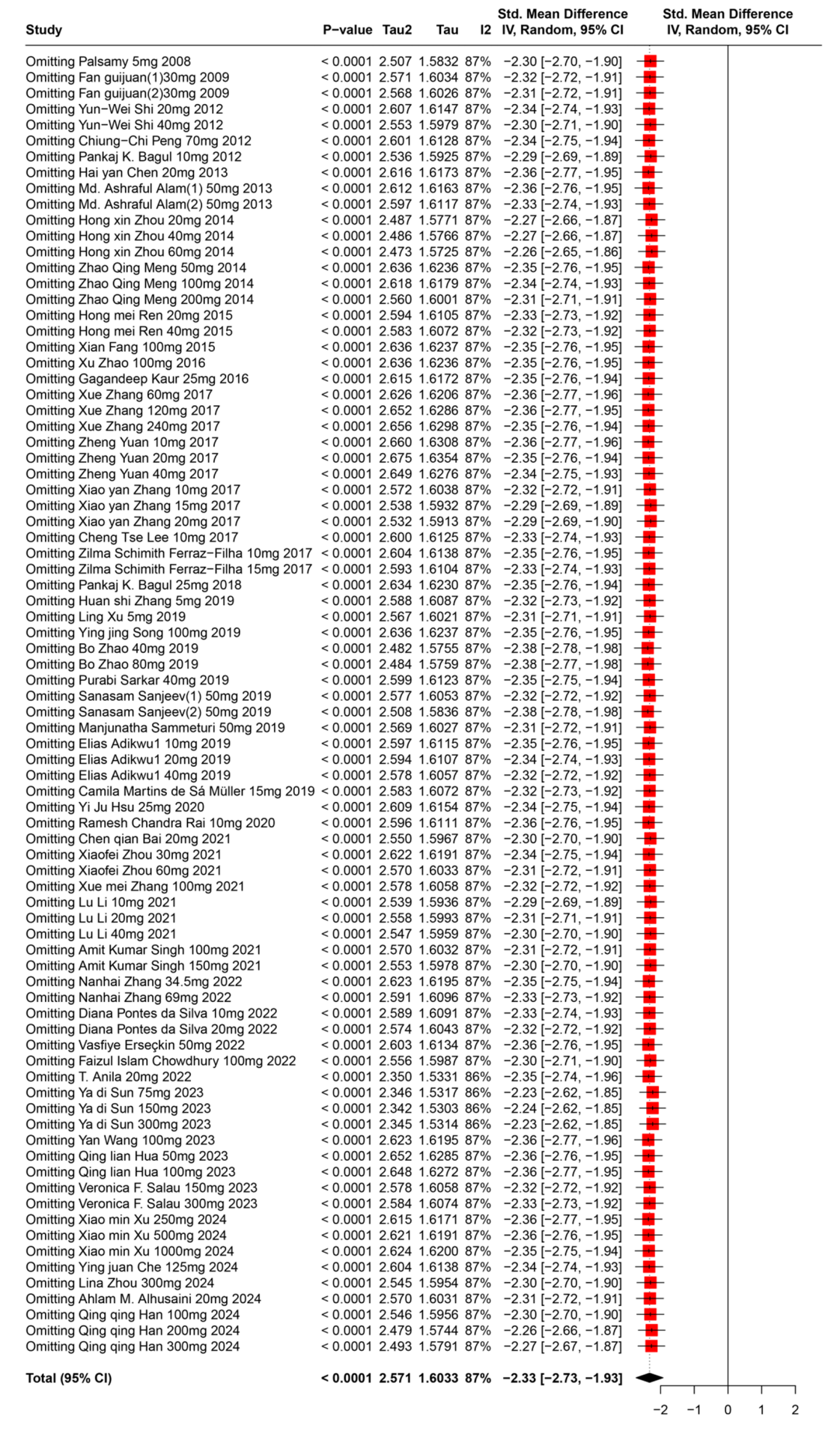


**Figure S1**.The effect of total polyphenols on SUA(sensitivity analysis)


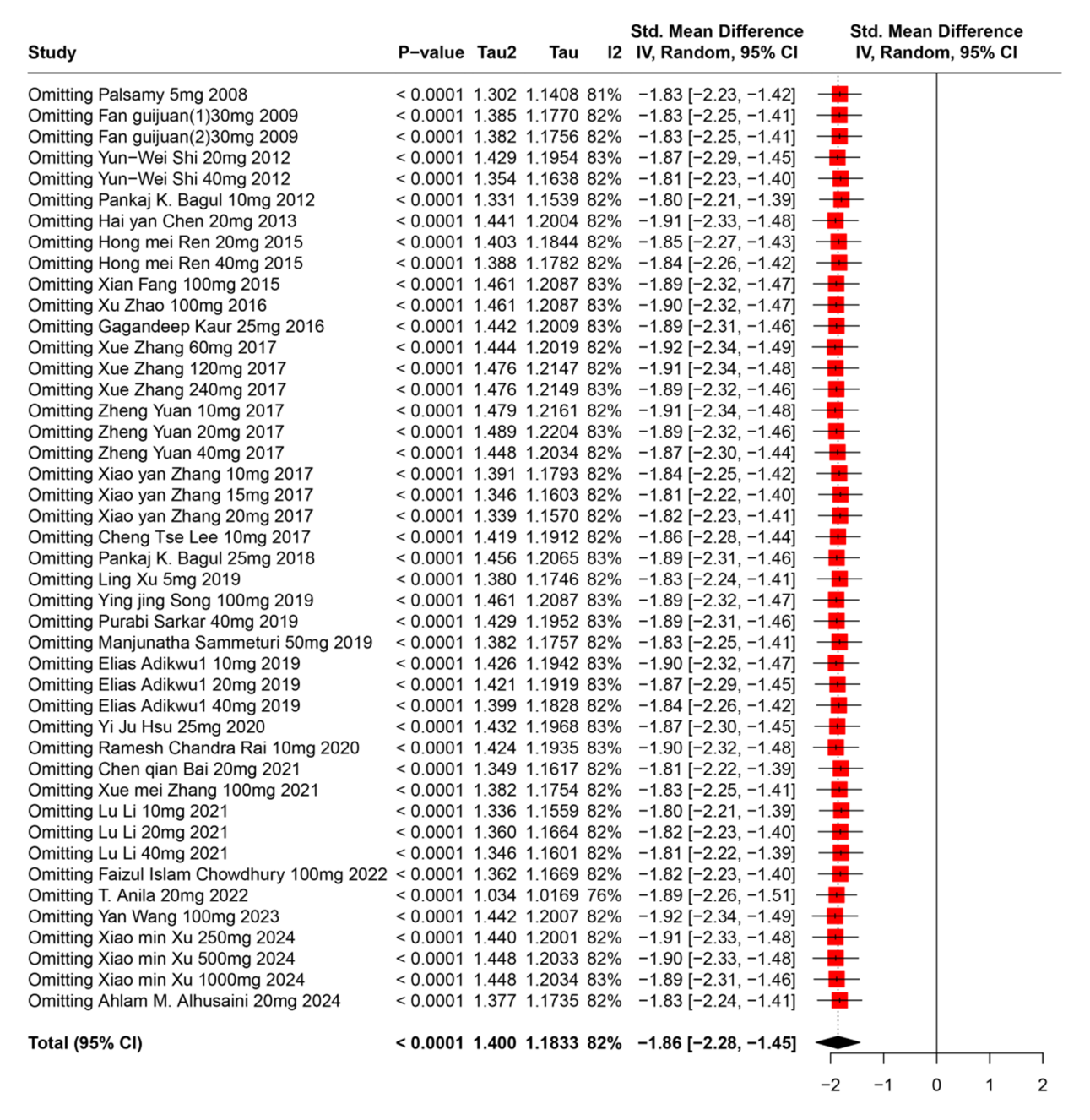


**Figure S2**. RES on SUA(sensitivity analysis)


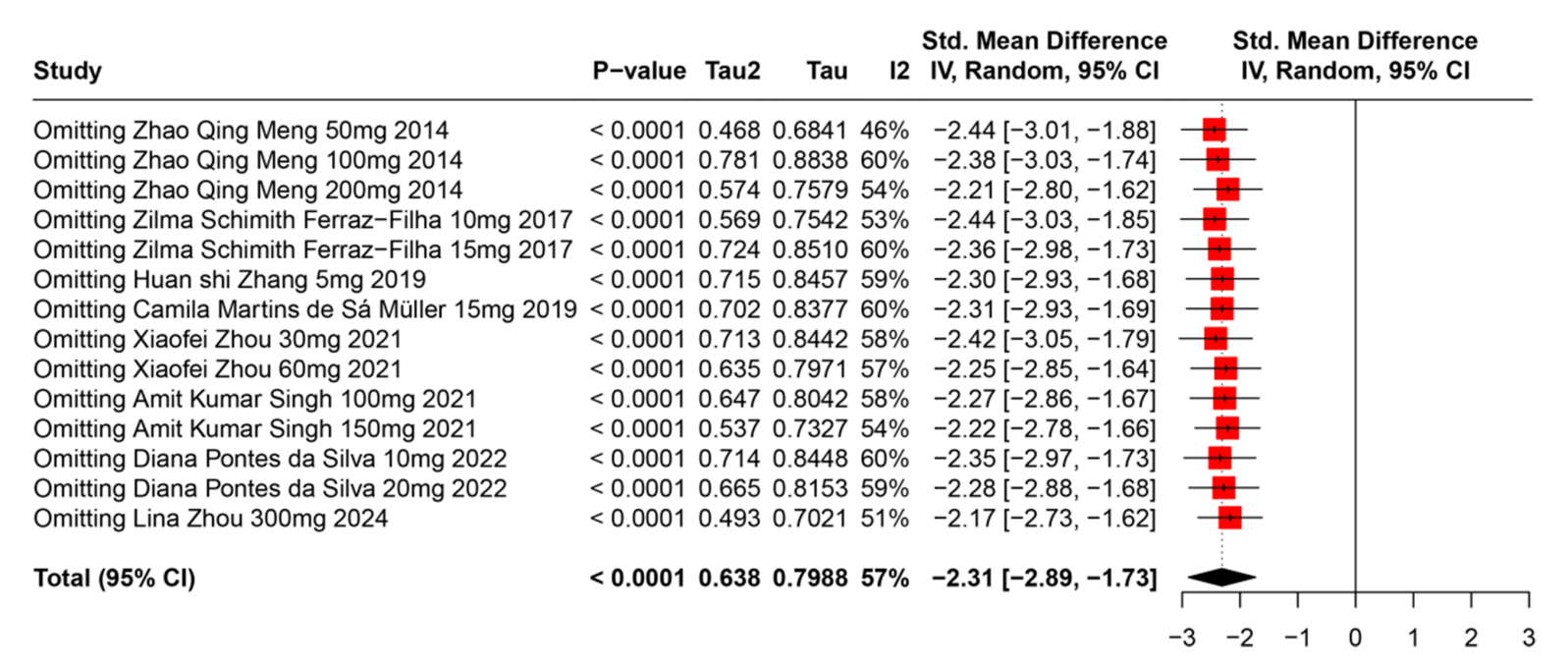


**Figure S3** CGA on SUA(sensitivity analysis)


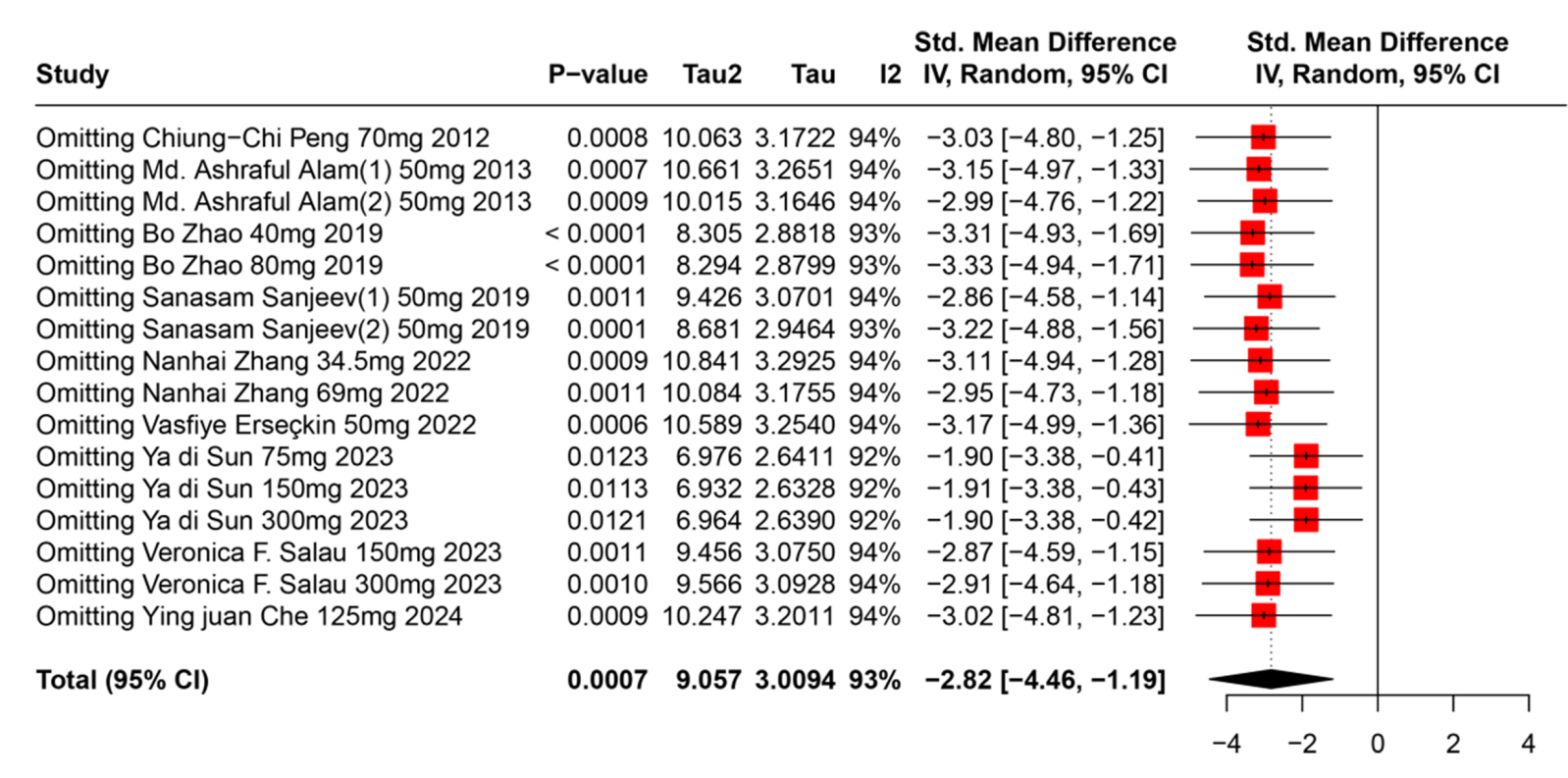


**Figure S4 FA on SUA(sensitivityanalysis)**


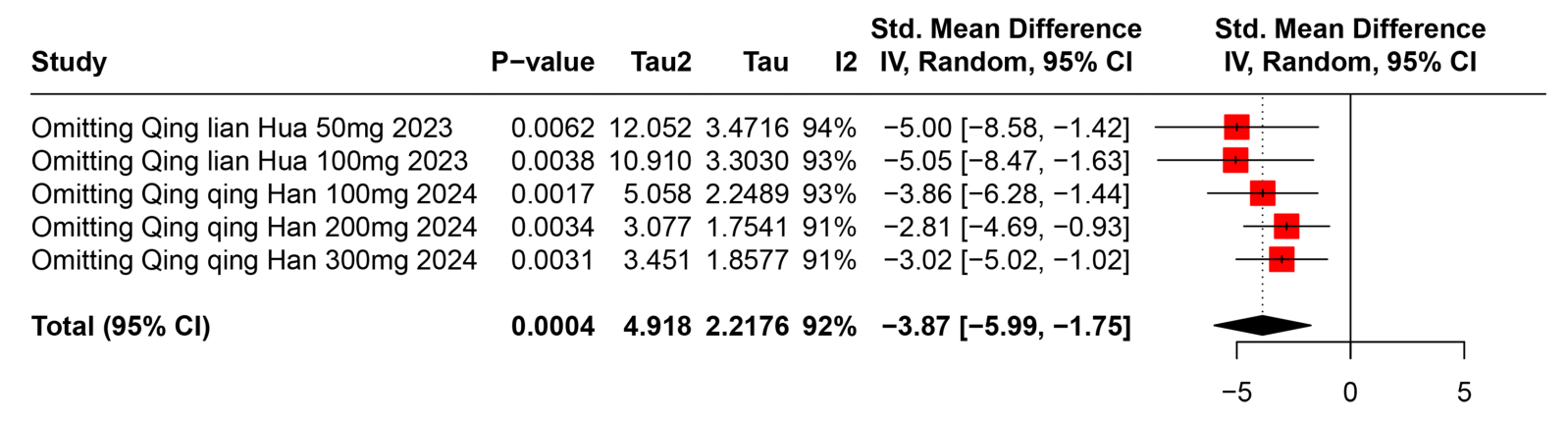


**Figure S5** PU on SUA(sensitivity analysis)


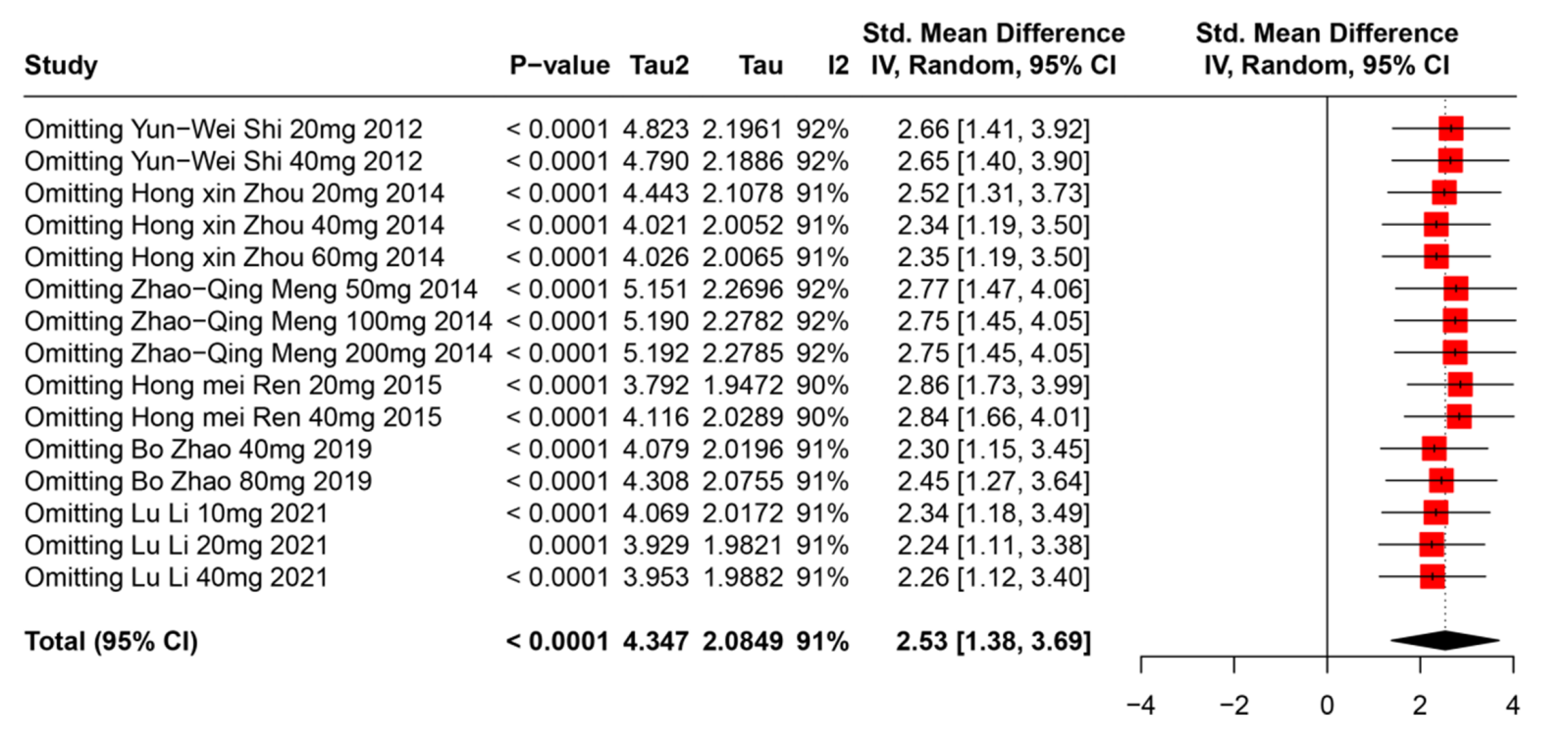


**Figure S6** The effect of total polyphenols on UUA(sensitivity analysis)


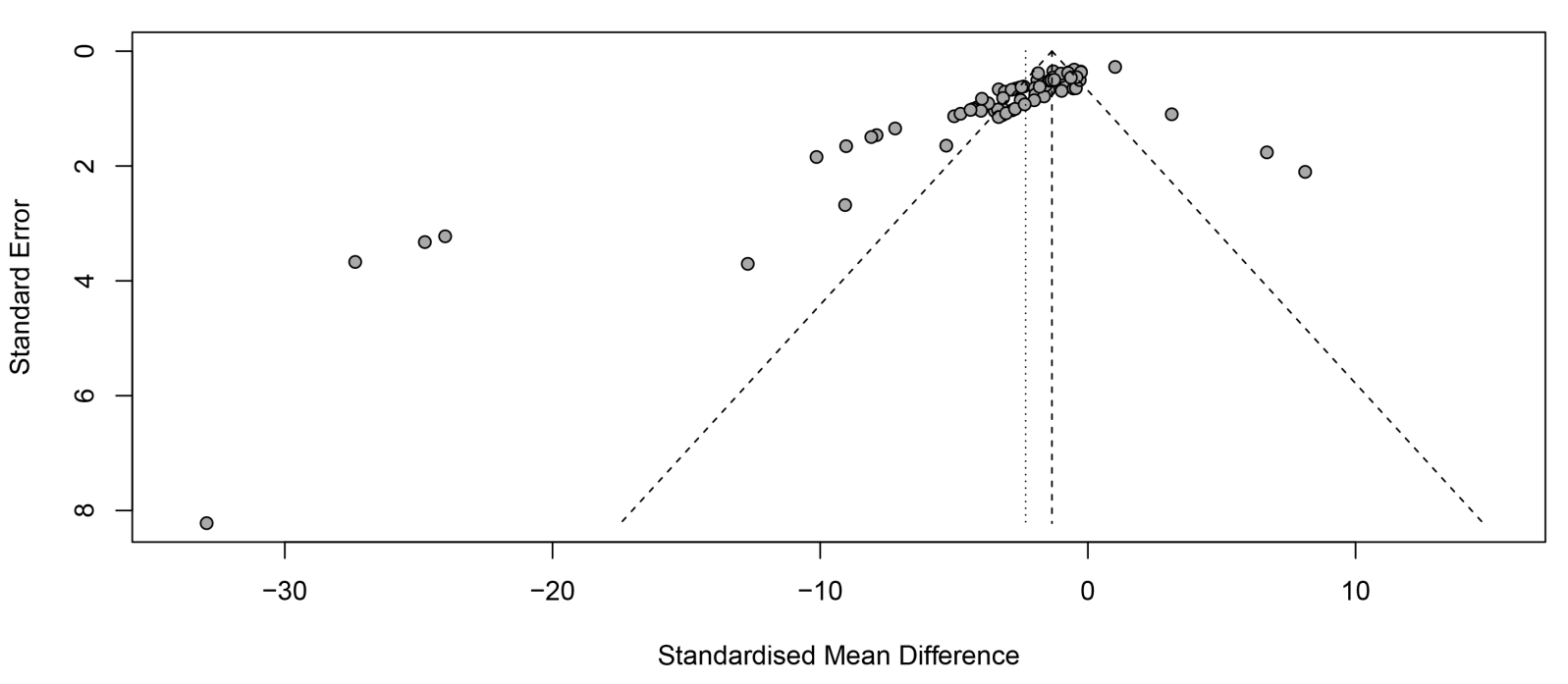


**Figure S7** Funnel plot of SUA after total polyphenolic compound intervention


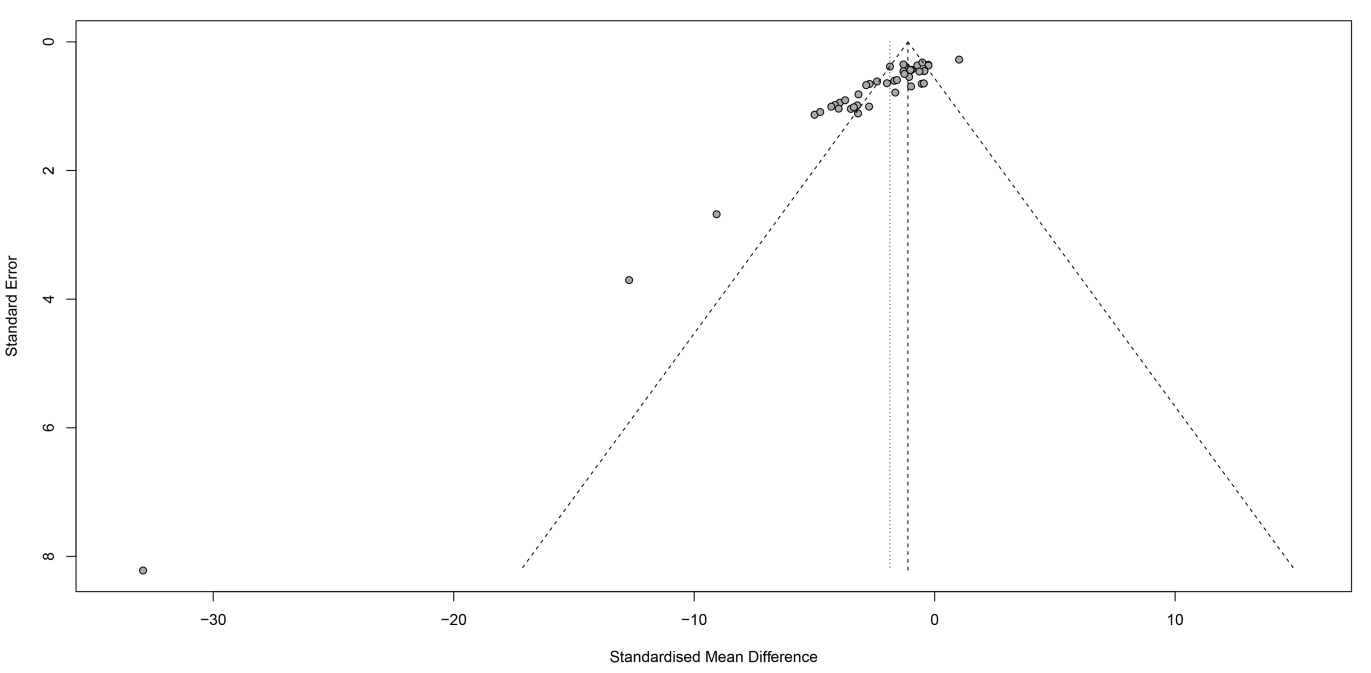


**Figure S8** Funnel plot of UUA after total polyphenolic compound intervention


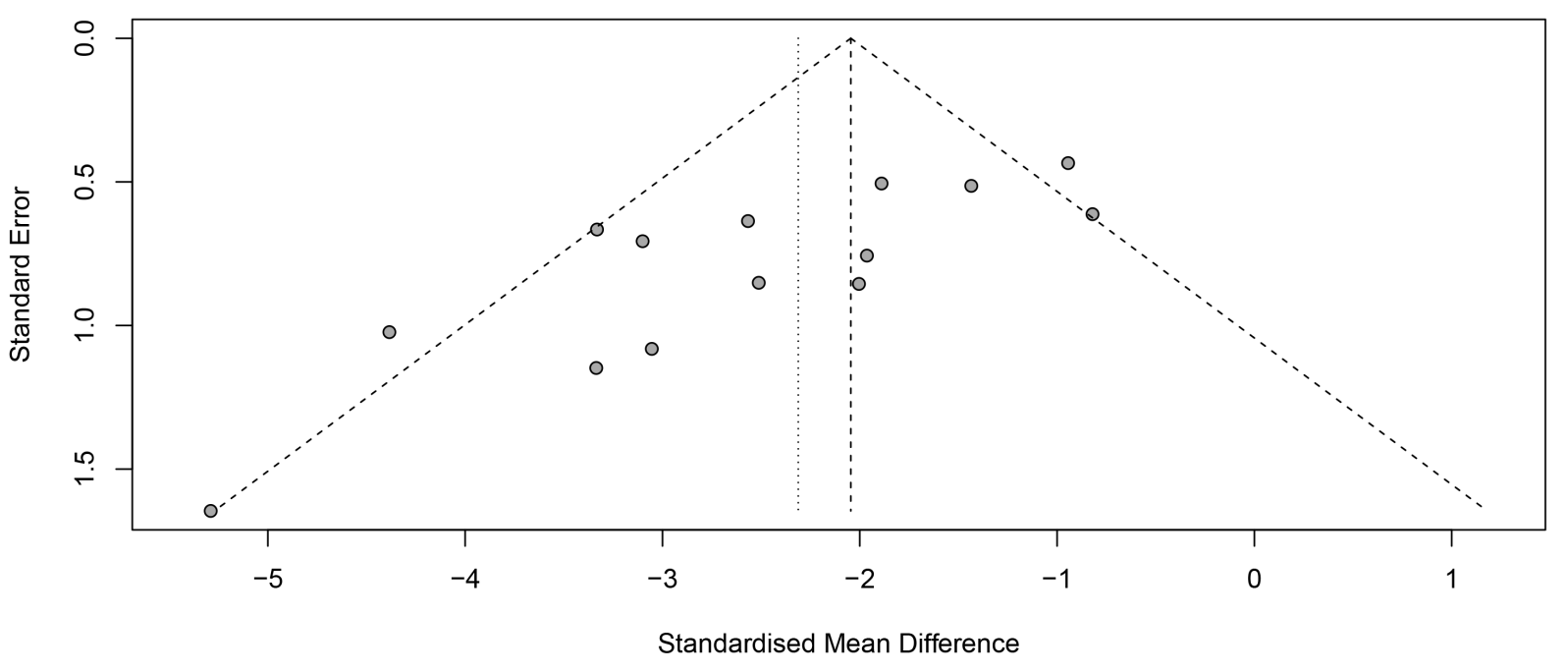


**Figure S9** Funnel plot of SUA after RES intervention


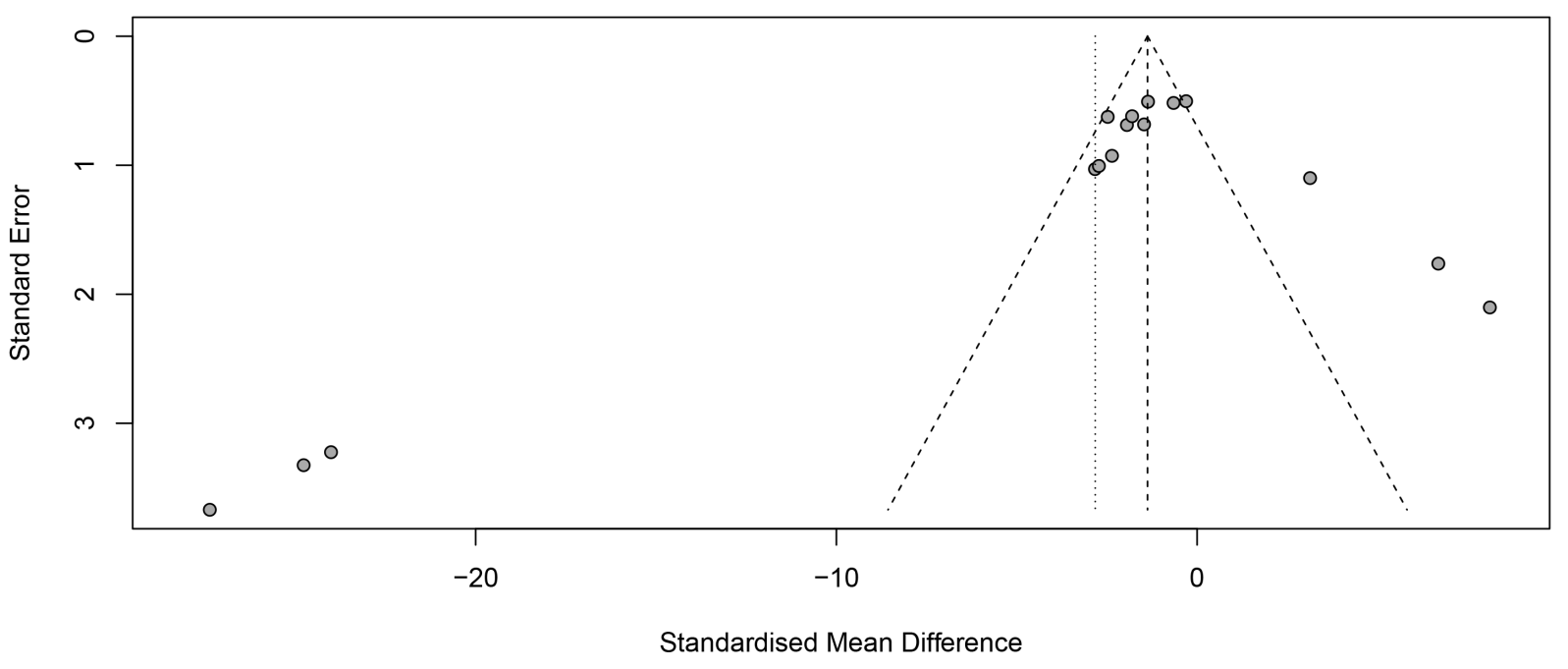


**Figure S10** Funnel plot of SUA after CGA intervention


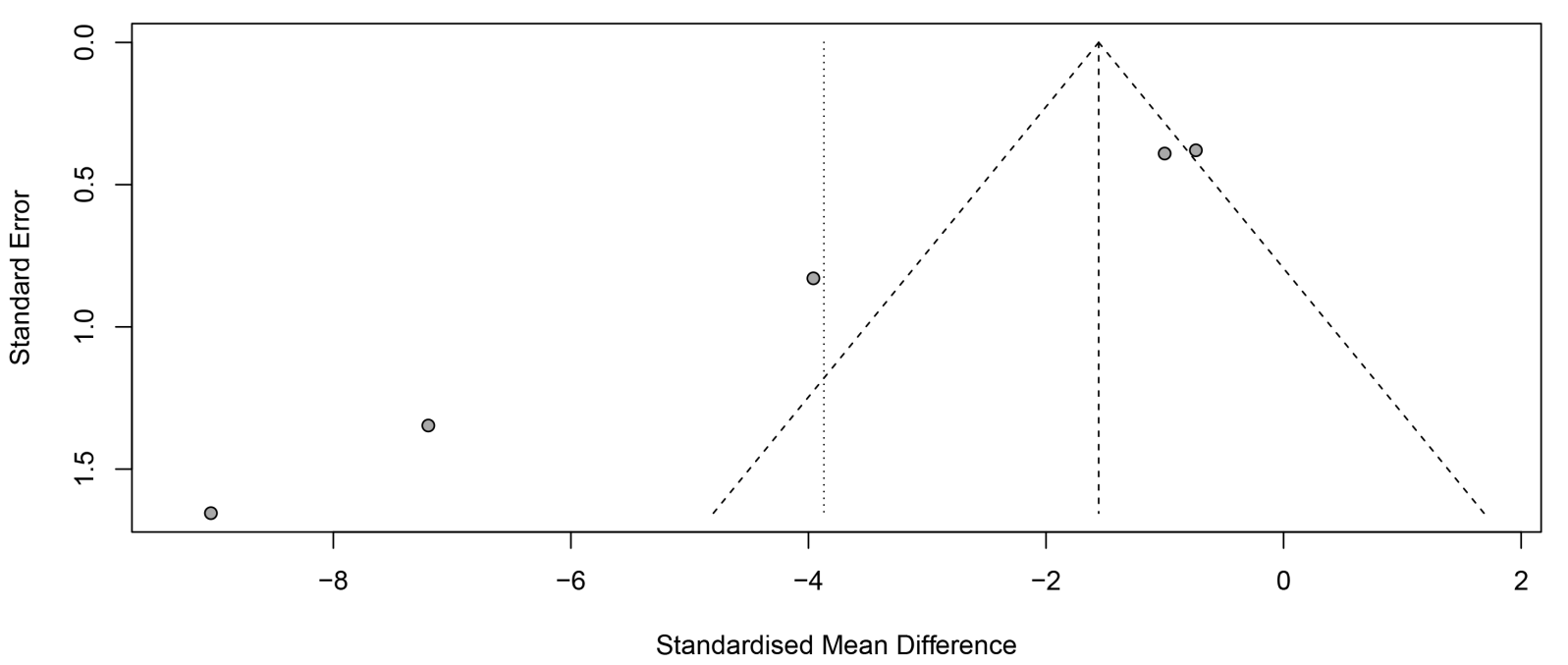


**Figure S11** Funnel plot of SUA after FA intervention


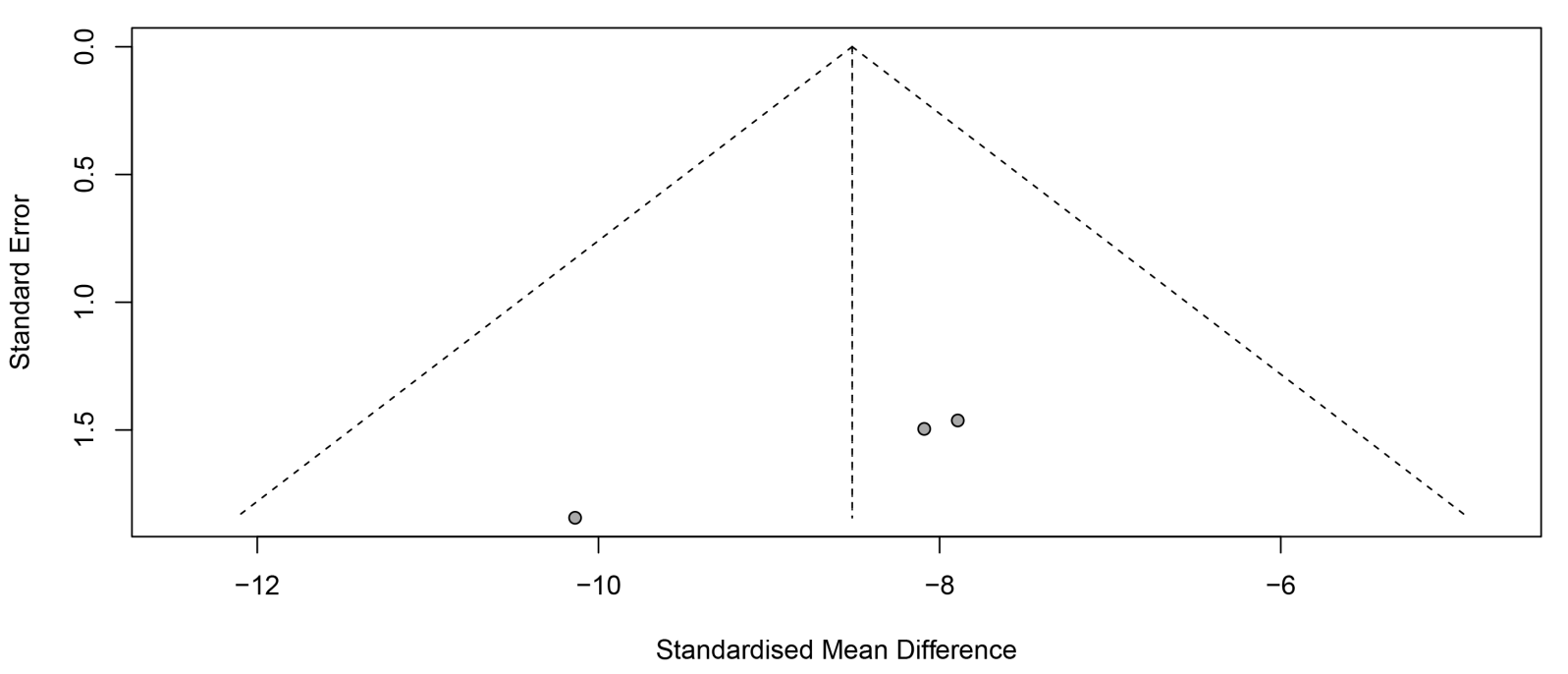


**Figure S12** Funnel plot of SUA after PU intervention


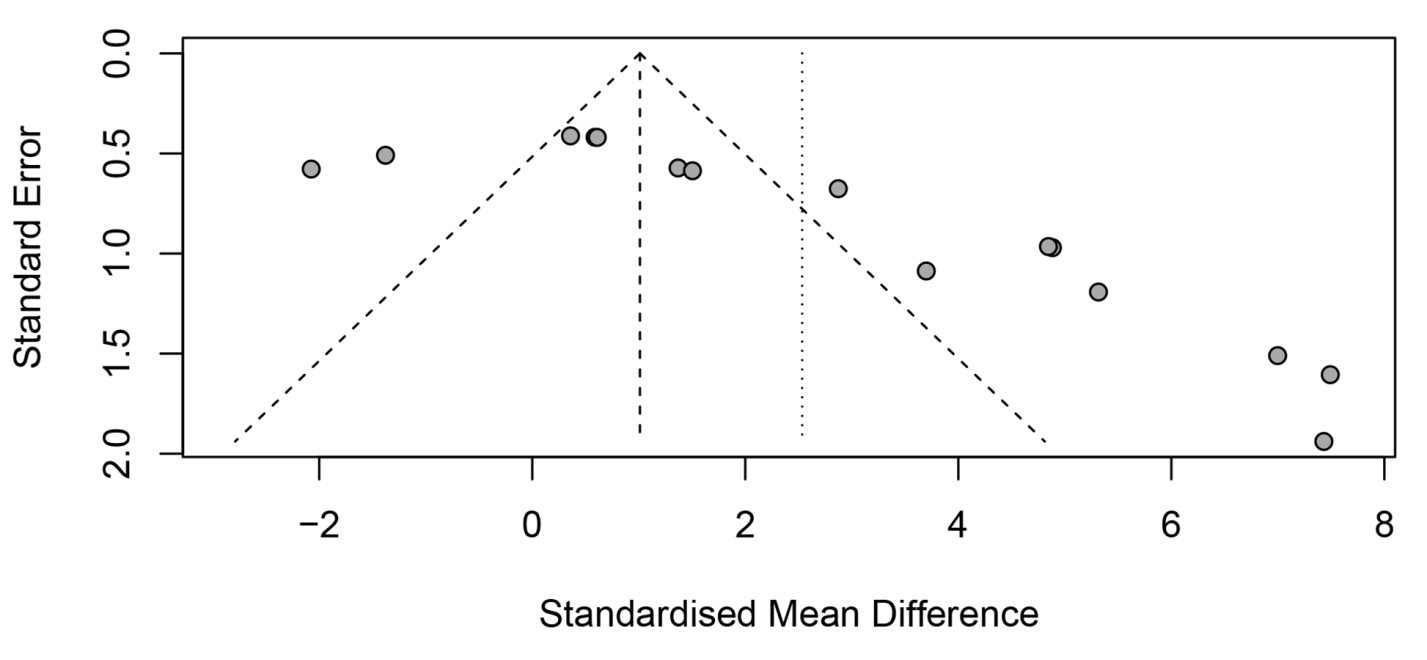


**Figure S13** Funnel plot of SUA after BER intervention
