# Supplementary material for: Urate-lowering effects of polyphenolic compounds in animal models: systematic review and meta-analysis
Source: PeerJ. 2025 Aug 11;13:e19731. doi: 10.7717/peerj.19731 (PMC12352422; doi:10.7717/peerj.19731)
Supplement: Supplemental Information 4 [file peerj-13-19731-s004.docx]

1. Target Audience

Basic Research Scholars:

Scientists engaged in natural product chemistry, pharmacology, nutrition, or biochemistry, focusing on the biological activities and mechanisms of polyphenols.

Applied Researchers:

Teams dedicated to developing drugs for hyperuricemia/gout, functional foods, or dietary supplements, requiring validation of the preclinical efficacy of polyphenols.

Preclinical Researchers:

Scientists specializing in translational research using animal models of human diseases, who need to systematically evaluate the experimental design and reliability of results regarding the uric acid-lowering effects of polyphenols.

Public Health and Policy Makers:

Individuals concerned with the potential of natural products in preventing and managing metabolic diseases, requiring scientific evidence to support updates to relevant guidelines or health policies.

2.Suitable Reading Groups

Academic Community:

Graduate students, postdoctoral researchers, and professors in the fields of metabolic diseases or natural products, who need to stay informed about the latest developments. Journal editors and reviewers who require a comprehensive understanding of the quality and limitations of evidence regarding the uric acid-lowering effects of polyphenols.

Industry:

Research and development departments of pharmaceutical companies or health supplement companies, who need to screen candidate compounds or optimize product formulations. Medical investment institutions that assess the market potential and scientific backing of polyphenol-based uric acid-lowering products.

Clinical and Educational Community:

Physicians specializing in rheumatology, immunology, or endocrinology, who need to update their knowledge on the use of natural products as adjunctive treatments for hyperuricemia. Medical school teachers who incorporate systematic review methods and case studies on polyphenols into their curricula.
